# Selective recognition of parallel and anti-parallel thrombin-binding aptamer G-quadruplexes by different fluorescent dyes

**DOI:** 10.1093/nar/gku833

**Published:** 2014-09-22

**Authors:** Dan Zhao, Xiongwei Dong, Nan Jiang, Dan Zhang, Changlin Liu

**Affiliations:** Key Laboratory of Pesticide and Chemical Biology, Ministry of Education and School of Chemistry, Central China Normal University, Wuhan 430079, China

## Abstract

G-quadruplexes (G4) have been found increasing potential in applications, such as molecular therapeutics, diagnostics and sensing. Both Thioﬂavin T (ThT) and N-Methyl mesoporphyrin IX (NMM) become fluorescent in the presence of most G4, but thrombin-binding aptamer (TBA) has been reported as the only exception of the known G4-forming oligonucleotides when ThT is used as a high-throughput assay to identify G4 formation. Here, we investigate the interactions between ThT/NMM and TBA through fluorescence spectroscopy, circular dichroism and molecular docking simulation experiments in the absence or presence of cations. The results display that a large ThT ﬂuorescence enhancement can be observed only when ThT bind to the parallel TBA quadruplex, which is induced to form by ThT in the absence of cations. On the other hand, great promotion in NMM fluorescence can be obtained only in the presence of anti-parallel TBA quadruplex, which is induced to fold by K^+^ or thrombin. The highly selective recognition of TBA quadruplex with different topologies by the two probes may be useful to investigate the interactions between conformation-specific G4 and the associated proteins, and could also be applied in label-free fluorescent sensing of other biomolecules.

## INTRODUCTION

Nucleic acids with four tracts of consecutive guanines are prone to forming a non-canonical four-stranded structure termed G-quadruplexes (G4). A G4 is characterized by the multilayered stack of planar G-quartets, each of which comprises four guanines and is stabilized by cyclic Hoogsteen hydrogen bonding (1,2). According to the number of G-rich strands and the orientation of oligonucleotides, the conformation of G4 has been differentiated into various families like inter- and intramolecular G4, or parallel, anti-parallel and a hybrid of parallel/anti-parallel G4 structures (3–6), which are affected by several factors, such as the number of stacked G-tetrads, the composition of loops (7,8), preparation protocol and the presence of diverse cations or small molecules (9–12). G4-forming oligonucleotides have attracted wide attention recently for their potential biological functions *in vivo* (13–24), as well as the application in label-free detection of other biomolecules through the G4-based fluorescent or luminescent probes (25–29). Efficient probing of G4 structures in living cells will promote the development of telomere-targeted anti-cancer therapy and quadruplex-specific medication. Furthermore, highly selective fluorescent recognition of G4 with exact topology is beneficial to study the interactions between G4-forming oligonucleotides and the associated proteins.

Recently, the benzothiazole dye Thioﬂavin T (ThT) has been reported as a fluorescence light-up probe for G-quadruplexes (30–33), which once was widely used in selectively staining and identifying amyloid ﬁbrils both *in vivo* and *in vitro* (34–36). It was proved by Jyotirmayee Mohanty *et al.* that ThT was capable of inducing the human telomeric sequence 22AG to form the parallel G4 in Tris-buffer and the anti-parallel G4 in water, respectively, both conditions produced much larger enhancements in ThT fluorescence compared with the presence of duplexes or single-strand DNA (30). Furthermore, Mergny's group demonstrated that the ThT fluorescence signal could be used to predict G4 formation, because great enhancement in ThT fluorescence could be obtained in the presence of most G4-forming sequences, but thrombin-binding aptamer (TBA) was the only exception, which led to a much lower increase in ThT ﬂuorescence compared with other G4-forming sequences (33).

Thrombin is a serine protease that plays an important role in thrombosis and hemostasis (37,38). The TBA, a single-stranded 15-mer DNA (5′-GGTTGGTGTGGTTGG-3′), was found to bind thrombin with high affinity and played important roles that inhibited the thrombin catalysed activity in the blood-clotting process (39,40). Actually, TBA was known to form a chair-like intramolecular anti-parallel G-quadruplex in the presence of thrombin or metal ions, such as K^+^ and Sr^2+^ (41,42). However, when two or four ‘T’ bases were mutated into ‘G’ in the lateral loop region of TBA, namely, TBA2G (5′-GGTGGGTGTGGTGGG-3′), the conformation of anti-parallel quadruplex could be changed completely to a parallel quadruplex in the presence of K^+^, which was reported by the teams of Modesto Orozco and Ramon Eritja recently (43).

In our study, we found that ThT was capable of inducing free TBA to fold into the parallel G4 conformation in the absence of cations, and gave rise to a great fluorescence signal simultaneously. However, very low promotion in ThT emission intensity could be observed when ThT reacting with the anti-parallel quadruplexes of TBA, which was induced to form by K^+^ or thrombin.

Another fluorescent probe, the unsymmetrical anionic porphyrin N-Methyl mesoporphyrin IX (NMM), has also been reported to exhibit a high selectivity for G4 DNA, but no significant binding activity for a variety of other nucleic acid species, including single-stranded DNA, duplex nucleic acids (duplex DNA and RNA, duplex DNA–RNA hybrids, Z-DNA) and triplex DNA (44–46). In our work, NMM did not show high affinity for the parallel TBA quadruplex which was folded in the presence of ThT alone, but exhibited a great fluorescence enhancement when the parallel TBA quadruplex was transformed into the anti-parallel quadruplex by thrombin or metal ions (K^+^ or Sr^2+^).

The study on the interactions between ThT and TBA in the absence or presence of K^+^ was an effective complement to the investigation of using ThT as a high-throughput assay to predict G4 formation and screen the G4-forming sequences. In addition, the specific recognition of the TBA quadruplex with different topologies by ThT and NMM, respectively, may be useful to investigate the interactions between conformation-specific G4 and the associated proteins, and could also be applied in label-free detection of other biomolecules, such as thrombin or biothiol (31).

## MATERIALS AND METHODS

### Oligonucleotides and chemicals

The oligonucleotides TBA (5′-GGTTGGTGTGGTTGG-3′) and TBA2G (5′-GGTGGGTGTGGTGGG-3′) used for the studies were synthesized and polyacrylamide gel electrophoresis purified by Invitrogen Technology (Shanghai, China); The fluorescently labeled oligonucleotides 5′-Cy5-TBA-3′, 5′-Cy3-TBA-3′, 5′-Cy5-TBA2G-3′ and 5′-Cy3-TBA2G-3′ were purchased from Takara Biotechnoly (Dalian). The concentrations of DNA were determined by ultraviolet (UV) spectrometry using extinction coefﬁcients provided by the OligoAnalyzer 3.1 from the web of INTEGRATED DNA TECHNOLOGIES.

ThT (3, 6-dimethyl-2-(4-dimethylaminophenyl) benzothiazolium cation) was purchased from Sigma-Aldrich, and NMM was obtained from Frontier Scientific (Logan, UT, USA). All other chemicals, such as SrCl_2_ and KCl, were purchased from China National Pharmaceutical Group Corporation as analytical grade. The concentration of NMM was calculated using the molar extinction coefﬁcient at 379 nm in water of 1.45 × 10^5^ M^−1^cm^−1^.

### Fluorescence scans

All fluorescence measurements were performed in 10 mM Tris-buffer, pH 7.2 on Cary Eclipse fluorescence spectrophotometer (Varian, USA) at 25°C. The ﬂuorescence emission of ThT was collected between 435 and 650 nm, and the excitation wavelength of ThT was set at 420 nm. While for NMM, the ﬂuorescence emission was collected from 555 to 750 nm, and the excitation wavelength was set at 400 nm. When the sample contains both ThT and NMM (the compound mixture), the excitation wavelength was set at 410 nm, and the emission spectra in the range of 435 ∼ 750 nm were recorded. In Fluorescence Resonance Energy Transfer (FRET) experiment, the ﬂuorescence emission was collected from 555 to 750 nm, and the excitation wavelength of Cy3 was set at 544 nm in wavelength.

### Circular dichroism (CD) measurements and melting curves

CD experiments were carried out on a Chariscan CD photomultiplier (Applied Photophsics Limited, UK) equipped with a Quantum Northwest TC125 temperature controller. The spectra were collected between 220 and 500 nm at 25°C in 10 mM Tris-buffer, pH 7.2 with a scanning speed of 100 nm/min and the response time was set to 0.2 s. A quartz cuvette with 1.0 cm path length was used for all spectra scanning samples. All of the CD spectra were baseline corrected for signal contributions from the buffer.

In thermal melting experiments, the samples mixed TBA (15, 30 and 45 μM, respectively) in 10 mM lithium cacodylate buffer (pH 7.2) with various concentrations of ThT (*r* = 10, 15, 20, 30 and 40, respectively, *r* = [ThT]/[TBA]) were heated at 95°C for 5 min, and cooled down slowly to room temperature, then incubated at 4°C for at least 6 h. The samples were detected using a quartz cuvette with 1.0 mm path length, and monitored at 265 nm from 10°C to 70°C at a heating rate of 0.5°C/min.

### Molecular docking simulation

Molecular docking of the 3D structure of anti-parallel quadruplex DNA (entry 1HAO in the Protein Data Bank) was carried out using the program suite AutoDock 4.2.0 (http://www.scripps.edu/mb/olson/doc/autodock). DNA structure was prepared for docking (47), and the structure of ThT or NMM was optimized using the SYBYL program. By using Autodock program suite, the flexible docking of ThT or NMM was done by the Lamarckian genetic algorithm, searching for favorable bonding conformations of the ligand at the sites of the target DNA (47,48).

The solvent molecules were removed from the DNA 3D structure to obtain the docking grid, and the active site was defined using Auto Grid. The grid size was set to 60*60*60 points with grid spacing of 0.375 Å, van der Waals well depth of 0.100 kcal/mol and iteration were set to 200 (49) and population size 100.

The graphical user interface AutoDockTools (ADT 1.4.6) was performed to setup each ligand-protein interaction, where all hydrogen atoms were added, Gasteiger charges were calculated and non-polar hydrogen atoms were merged to carbon atoms. The best ranked pose was selected from the ChemScore. The conformation with the lowest binding energy was used to analyze ligand placement.

## RESULTS AND DISCUSSION

### Efficient fluorescent recognition of the parallel TBA quadruplex by ThT

We studied the interactions between ThT and TBA in the free state through UV, fluorescence, CD and molecular docking experiments, and the results indicated that ThT was capable of inducing TBA to form the parallel quadruplexes with strong enhancement in fluorescence in the absence of cations.

**Figure 1.**
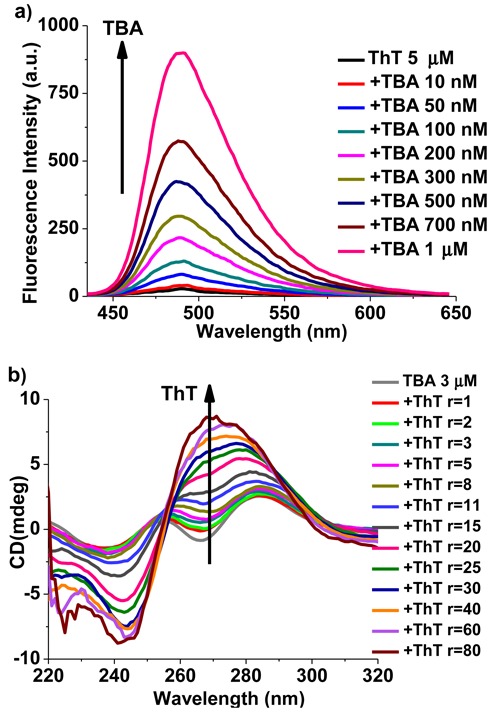
(a) Fluorescence titration of ThT (5 μM) with various concentrations of TBA, λ_ex_ = 420 nm. The arrow from bottom to top represents increments of TBA from 0 to 1 μM. (b) CD titration spectra of TBA (3 μM) with ThT in the absence of any metal ions. The arrow from bottom to top indicates increments in *r* value from 0 to 80. (*r* = [ThT]/[TBA]).

ThT dye displayed its characteristic absorption profile with maximum at 414 nm when diluted in pH 7.2 Tris-buffer (Supplementary Figure S1). Upon the addition of unfolded TBA, obvious bathochromic shifts (from 414 to 436 nm) and hyperchromic effects were observed, which were similar to the results of titration of TBA with human telomeric sequence 22AG (30). The strong binding between ThT and TBA was further studied by monitoring the fluorescence intensity of ThT (Figure 1a). When excited at the isosbestic points (∼420 nm), 5 μM ThT presented very weak emission at 487 nm in Tris-buffer, and a dramatic fluorescence enhancement was obtained after the increased addition of TBA (up to 1 μM), indicating the selective fluorescence recognition of TBA by ThT in the absence of cations.

The induced circular dichroism (ICD) spectra (Figure 1b) exhibited that unfolded TBA had weak positive bands around 255 and 285 nm, as well as weak trough near 265 nm in the absence of metal ions. Upon titration with ThT, a positive band at ∼265 nm as well as a negative band at ∼240 nm appeared gradually, indicating the formation of parallel G4 structures (43).

We further confirmed the formation of intermolecular parallel quadruplexes of TBA induced by ThT through FRET experiments (Supplementary Scheme S1). In this assay, Cy3 (λex = 544 nm, λem = 566 nm) and Cy5 (λex = 649 nm, λem = 667 nm) were used as the donor and acceptor fluorophores to label the 5′ terminus of TBA or TBA2G. When TBA folded into anti-parallel G-quadruplexes in the presence of K^+^, the two fluorophores, Cy3 and Cy5, were separated from each other. But when it folded into intermolecular parallel G-quadruplexes, it would be possible for the two fluorophores to be adjacent to each other and then carry out FRET.

We titrated the mixed solution of labeled TBA (Cy3-TBA: Cy5-TBA = 1:1) with ThT under the excitation of 544 nm light. Upon the addition of ThT, the emission maximum of Cy3 at 566 nm decreased, and the emission maximum of Cy5 at 667 nm increased simultaneously (Supplementary Figure S2a), which indicated the formation of intermolecular quadruplexes. The results of control experiments displayed that there was no increase at 667 nm when titrating the mixture of Cy3-TBA and Cy5-TBA with K^+^, which was due to the formation of parallel TBA quadruplexes (Supplementary Figure S2b). And ThT was also capable of quenching the fluorescence of Cy3, but did not increase the intensity of emission around 667 nm (Supplementary Figure S2c). Besides, as the modified sequence TBA2G has been reported to form the intermolecular parallel quadruplexes in the presence of K^+^ (43), the phenomenon of FRET could also be observed when titrating the labeled TBA2G (Cy3-TBA2G: Cy5-TBA2G = 1:1) with K^+^ (Supplementary Figure S2d). Therefore, it is reasonable for us to conclude that ThT was capable of inducing TBA to form intermolecular parallel quadruplexes.

Moreover, the extent of quadruplex stabilization in the presence of ThT was assessed from the melting temperature (*T*_m_), which was evaluated from the CD heat-denaturation profiles. The melting curves of the parallel quadruplex were monitored at 265 nm. In the absence of metal ions, TBA was in the unfolded state and no stable structure could be monitored. When mixed with various equiv of ThT, TBA was induced to form a parallel quadruplex and became more stable with the increased addition of ThT. The melting curves of TBA (15, 30 and 45 μM, respectively) with various equiv of ThT (*r* = 10, 15, 20, 30 and 40, respectively) were shown in Supplementary Figure S3, and three fitting lines were obtained from plotting *T*_m_ against the concentration ratio ‘*r*’ (Supplementary Figure S3d), the simultaneous increase of TBA concentration and *T*_m_ confirmed the intermolecular character of G4 folding. Overall, these results indicated that ThT could induce TBA to fold into a parallel quadruplex in pH 7.2 Tris-buffer or lithium cacodylate buffer. For water solution, ThT could also induce TBA to form a parallel quadruplex ultimately with strong fluorescence emission (data were shown in Supplementary Figure S4). Therefore, it is reasonable to conclude that the selective fluorescent recognition of TBA by ThT was attributable to the strong interactions between ThT and the parallel TBA quadruplex.

We further examined the fluorescence intensity provided by the reaction of ThT with the anti-parallel TBA quadruplex, which had been reported to form in the presence of K^+^ or Sr^2+^ (41,42). For both TBA alone and the mixture of TBA and ThT, a peak at ∼295 nm as well as a trough at ∼265 nm (anti-parallel folding) appeared with addition of K^+^ (Supplementary Figure S5a and b), indicating that K^+^ could not only induce TBA to form the anti-parallel conformation, but also transform the pre-folded parallel quadruplex into anti-parallel quadruplex. The fluorescence spectra displayed a remarkable decrease in the emission intensity with the increase of K^+^ (Supplementary Figure S5c), which was believed to be due to the structure transition of TBA from the parallel to anti-parallel quadruplex. In this respect, ThT could be used as a specific fluorescence probe for the parallel TBA quadruplex.

**Figure 2.**
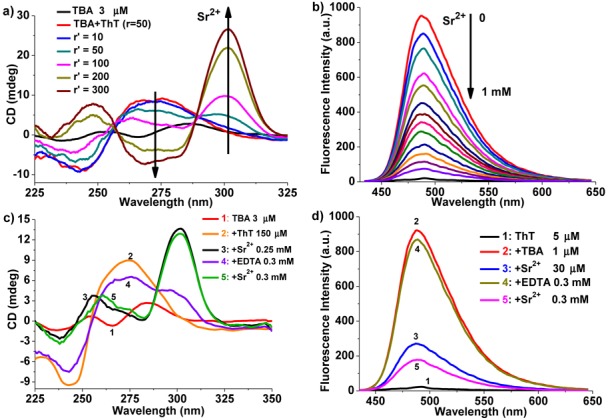
(a) CD titration of TBA (3 μM) with various concentrations of Sr^2+^ in the presence of ThT (150 μM). The arrows indicate increments in *r*′ value from 0 to 300. (*r*′ = [Sr^2+^]/[TBA]). (b) Fluorescence titration of TBA (1 μM) and ThT (5 μM) with Sr^2+^. The arrow from top to bottom indicates the addition of Sr^2+^ from 0 to 1 mM. The bottom black spectrum curve is the background signal of ThT. λ_ex_ = 420 nm. (c and d) Cycling of the Sr^2+^-mediated structural conversion of TBA monitored by CD (c) and fluorescence (d) in the presence of ThT.

**Scheme 1.**
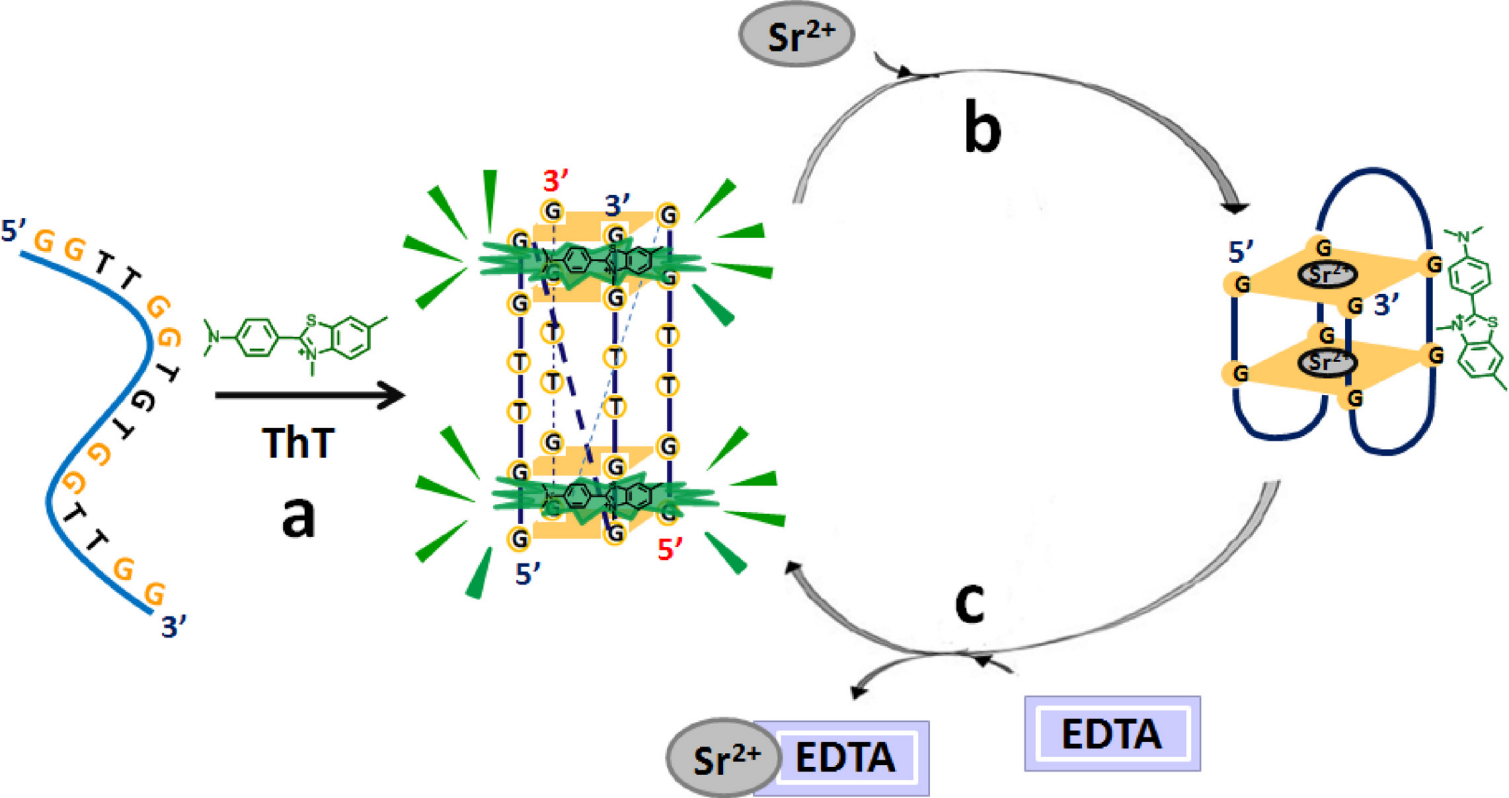
Structural conversion between the parallel and anti-parallel TBA quadruplexes mediated by ThT and Sr^2+^. (a) ThT induces TBA to form the parallel quadruplex and produces strong fluorescence emission. (b) The transformation of the parallel quadruplex into anti-parallel quadruplex upon addition of Sr^2+^, along with a remarkable decrease in the emission intensity of ThT. (c) Regeneration of the parallel TBA quadruplex and the strong fluorescence signal by ThT upon the removal of Sr^2+^ through EDTA.

Scheme 1 showed the results as mentioned above between the changes in ThT fluorescence and the structural conversion of TBA, which was mediated by the addition of Sr^2+^ and ethylenediaminetetraacetic acid (EDTA). The ICD spectra results (Supplementary Figure S6) exhibited that Sr^2+^ induced TBA to form the anti-parallel quadruplex with a peak at ∼300 nm and a trough at ∼275 nm, which was in agreement with the reported results (42). Meanwhile, the addition of EDTA would release pre-folded anti-parallel quadruplex through removing Sr^2+^ from the folded structure. Therefore, ThT, Sr^2+^ and EDTA were used to mediate the topological structure of TBA. When titrating the mixture of TBA and 50 equiv of ThT with Sr^2+^, a positive band at ∼300 nm appeared and grew gradually (Figure 2a), whereas the positive band near 275 nm decreased and became a negative band, indicating that Sr^2+^ could also transform the pre-folded parallel quadruplex into the anti-parallel quadruplex, and then led to the dramatic decrease in the emission intensity of ThT (Figure 2b). However, when EDTA was subsequently added into this solution, the structure of the anti-parallel quadruplex was destroyed (Figure 2c) and the emission intensity of ThT recovered due to the regeneration of the parallel quadruplex (Figure 2d). Therefore, a cycling phenomenon could be obtained through adding Sr^2+^ and EDTA alternately into the mixture of TBA and ThT, which was exhibited in Scheme 1 and Figure 2c and d.

### Binding mechanism

**Figure 3.**
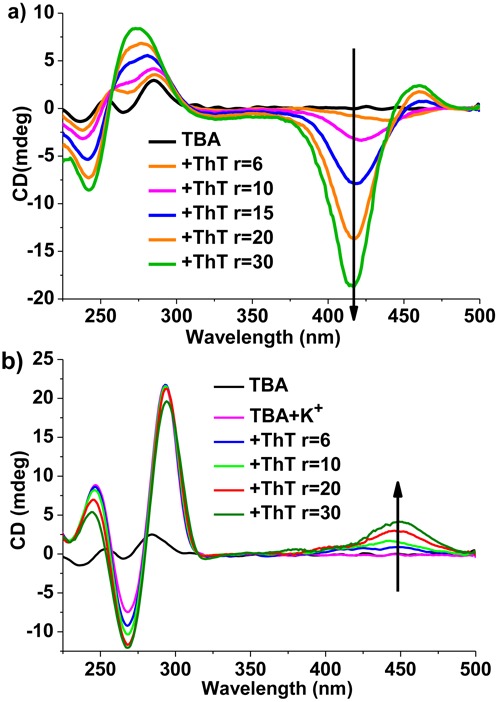
CD spectra recorded for TBA (3 μM) upon addition of various concentrations of ThT in the absence (a) and presence (b) of K^+^ (10 mM) in 10 mM Tris-buffer, pH 7.2. The arrows indicate increments in *r* value from 0 to 30. *r* = [ThT]/[TBA].

The binding mechanism between ThT and the parallel or anti-parallel quadruplex of TBA were studied by ICD spectra and molecular docking simulation experiments to further explain the differences in ThT fluorescence intensity. As reported by Jyotirmayee Mohanty *et al.*, when ThT bound to G4 DNAs, the binding mode between ThT and G4 could be analyzed from the absorption region of ThT (350 ∼ 500 nm) in ThT-TBA ICD spectra (30), that is, a positive ICD signal in the wavelength range of 350 ∼ 500 nm could be taken as an indicator of the groove binding to G4 structures (30,50), while a negative ICD band in the same region could be presumed as an intercalation mode (51). In our study, when ThT was added to TBA solutions in the absence of cations, the ICD spectra (Figure 3a) displayed that in the formation of parallel quadruplex (seen from the region below 350 nm), a strong negative band around 415 nm as well as a weak positive band near 460 nm appeared in response to the addition of ThT, indicating that ThT bound to the parallel quadruplex of TBA mainly in the mode of intercalation, and then gave rise to much stronger emission enhancement. In another case, when titrating TBA with ThT in the presence of enough K^+^, the folded anti-parallel quadruplex of TBA kept unchanged (seen from the region below 350 nm of Figure 3b), whereas a moderate positive ICD band appeared around 450 nm, indicating a typical groove binding mode of ThT to the anti-parallel quadruplex structures.

**Figure 4.**
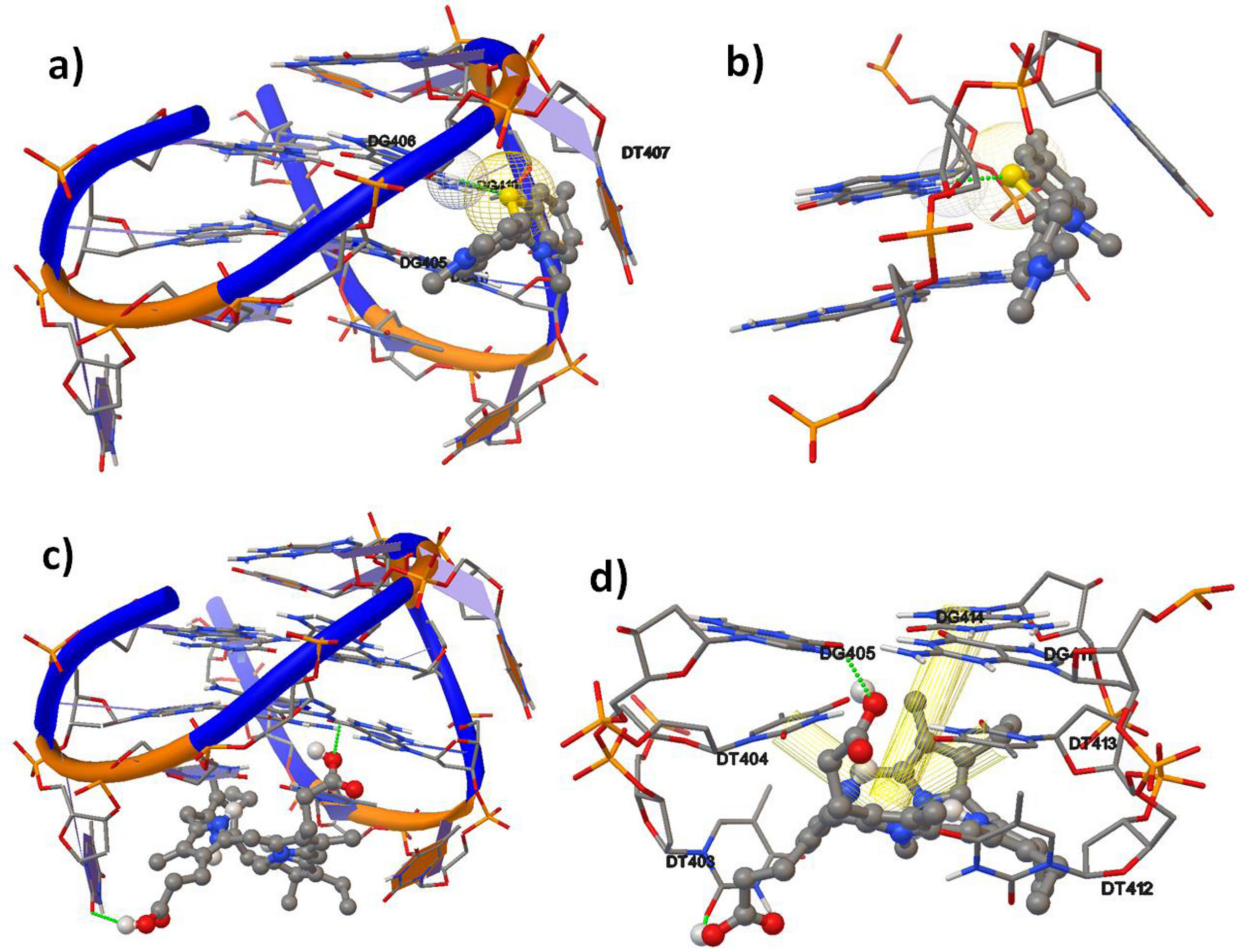
The interactions between the anti-parallel quadruplex (PDB: 1HAO) and ThT (a and b) or NMM (c and d) visualized by molecular docking simulation. DNA and ThT are represented as cartoon sticks and spheres. C, gray; N, blue; S, yellow; O, red. Green dotted lines represent the hydrogen bonding between bases and ThT/NMM, and yellow cylinders represent π∼π interactions between bases and NMM. (a) Side view of the anti-parallel TBA quadruplex with ThT. (b) The interactions of ThT with two quartets in the region of groove binding. (c) Side view of the anti-parallel TBA quadruplex with NMM. (d) The interaction of NMM with the bottom quartet as well as two T loops.

As no information on the crystal structure of parallel TBA quadruplex could be obtained from PDB now, the molecular docking simulation was carried out to further understand the interactions between the chair-like intramolecular anti-parallel TBA quadruplex (PDB ID: 1HAO) and energetically optimized ThT, using the program suite Auto Dock 4.2.0 (52–54) and SYBYL. The binding mode with the lowest binding energy and the most favorable conformation (Figure 4a) showed that ThT bound to the anti-parallel quadruplex only in the groove region composed by G_5_, G_6_, G_10_ and G_11_ of TBA, with a strong hydrogen bond between G_6_ and the atom S of ThT (Figure 4b), which was in agreement with the results of ICD spectra.

As the fluorescence of ThT was strongly influenced by its conformational and electronic properties, and the changes in ThT emission intensity were mainly associated with the angle between the two conjugated aromatic rings of ThT, benzothiazole and dimethylaminobenzene (34–36). It is reasonable to presume that, compared with the groove binding mode, the intercalation mode was beneficial to immobilizing ThT at a right angle and preserve the excited state, resulting in the great emission enhancement.

### Specific fluorescent recognition of the anti-parallel TBA quadruplex by NMM

In our work, we found that NMM displayed efficient fluorescent recognition of the anti-parallel TBA quadruplex, but did not show a high affinity with the parallel G4 conformation, which was folded in the presence of ThT alone. The results of ICD spectra and fluorescent titration experiments displayed that neither new ICD spectra of TBA nor emission enhancement of NMM could be observed when TBA reacted with NMM (Supplementary Figure S7a and b), indicating that NMM could not induce free TBA to form any kind of G4 in the absence of cations. However, when K^+^ or Sr^2+^ ions were subsequently added into the mixture of NMM and TBA, TBA was induced to form the anti-parallel quadruplex and then was recognized by NMM with great emission enhancement in the region of 600 ∼700 nm (Supplementary Figure S7c and d).

To gain further insight into the selective binding of NMM toward the anti-parallel TBA quadruplex, the molecular docking simulation was also used to dock energetically optimized NMM and the anti-parallel TBA quadruplex 1HAO. The most optimal binding mode (Figure 4c) demonstrated that NMM stacked at the bottom quartet of the anti-parallel TBA qudruplex in a non-planer form, and bound to the region of two T loops of G4. As displayed in Figure 4d, there were two hydrogen bonds between G_5_ or T_3_ and the carboxyl group of NMM, which was indicated by green dotted lines. Moreover, there were strong π∼π interactions between T_4_ or T_13_ and NMM (55), as well as weak π∼π interactions between NMM and G_14_ (56), which were indicated by yellow cylinders. In this case, it is judicious to presume that the efficient fluorescent recognition of the anti-parallel TBA quadruplex by NMM was mainly attributed to the binding of the two T loops. While in the parallel quadruplex of TBA, the two T loops were separated from each other, and thus made it hard for NMM to bind with the parallel G4.

### The specificity of the two probes toward TBA quadruplexes with different topologies

**Figure 5.**
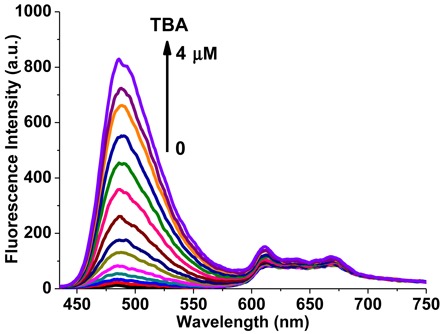
Fluorescence spectra of the mixture of 5 μM ThT and 10 μM NMM upon titration with TBA. The arrow from bottom to top represents increments of TBA from 0 to 4 μM. (λ_ex_ = 410 nm).

The former study demonstrated that the emission enhancement of ThT or NMM was only obtained in the presence of responding parallel or anti-parallel TBA quadruplex, respectively. As ThT and NMM have close excitation peak wavelengths (420 nm for ThT and 399 nm for NMM), and well-separated emission wavelengths (487 nm for ThT and 610 nm for NMM), we mixed the two compounds together to evaluate their specificity toward the parallel or anti-parallel quadruplex, which was mediated by metal ions or thrombin. When the mixture of 5 μM ThT and 10 μM NMM were excited with light of 410 nm, the fluorescence spectra displayed a very weak emission profile around 487 nm as well as the region of 600 ∼ 700 nm for ThT and NMM, respectively (Figure 5). However, gradual addition of TBA (up to 4 μM) increased the fluorescence intensity of ThT, while had almost no effect on the emission intensity of NMM, which could be explained by the formation of the parallel TBA quadruplex induced by ThT and the strong affinity between them. Furthermore, when various concentrations of Sr^2+^ or K^+^ were subsequently added into this sample, the parallel quadruplex of TBA was transformed into the anti-parallel conformation, resulting in the decrease in ThT fluorescence intensity as well as great enhancement in NMM emission, due to the release of ThT and the binding of NMM to the anti-parallel TBA quadruplexes (Supplementary Figure S8).

Thrombin was known to induce TBA to form the anti-parallel quadruplex and bind to this structure with high affinity. In our study, we found that thrombin could also transform the pre-folded parallel quadruplex into the anti-parallel conformation (Supplementary Figure S9a), which was similar to those caused by K^+^ or Sr^2+^. Therefore, in the solution of ThT and TBA, the remarkable decrease in ThT emission intensity was observed along with the addition of thrombin (Supplementary Figure S9b). On the other hand, the great enhancement in NMM emission was obtained through the titration of the solution of NMM and TBA with thrombin (Supplementary Figure S9c). Although the bio-macromolecule thrombin might produce a strong steric hindrance, it should be noted that the binding of thrombin and TBA might have no discernible effect on the interaction between NMM and the anti-parallel quadruplex of TBA.

**Figure 6.**
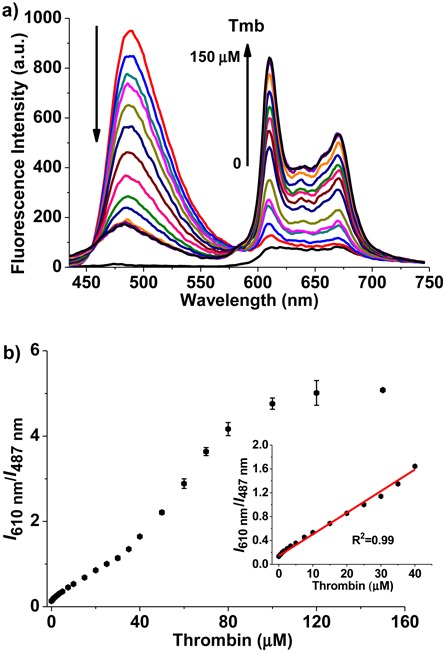
(a) Fluorescence spectra of the two compounds mixed together in the presence of TBA (4 μM) upon addition of thrombin. The bottom black spectrum curve is the background signal of the mixture of ThT and NMM. The arrows indicate increments in thrombin (Tmb) concentrations from 0 to 150 μM. (b) Plot of the NMM/ThT emission intensity ratio (*I*_610 nm_/*I*_487 nm_) against the concentration of thrombin. Insert: A linear response of *I*_610 nm_/*I*_487 nm_ versus thrombin concentration was observed over the range of 0∼40 μM (*R*^2^ = 0.99). All titration were carried out in 10 mM Tris-buffer, pH 7.2. (λ_ex_ = 410 nm).

The specificity of the two fluorescent probes toward the parallel or anti-parallel TBA quadruplex could also be confirmed by thrombin in the absence of cations. When thrombin was added into the mixed solution of 5 μM ThT, 10 μM NMM and 4 μM TBA, the fluorescence intensity of ThT centered at 487 nm decreased, whereas the fluorescence intensity of NMM at 610 nm increased (Figure 6a). Plotting the relative fluorescence intensity (*I*_610 nm_/*I*_487 nm_) against the concentration of thrombin, a linear response of *I*_610 nm_/*I*_487 nm_ versus thrombin concentration showed over the range of 0∼40 μM with a correlation coefﬁcient (*R*) of 0.99 (Figure 6b). In this case, the limit of detection (at a signal-to-noise ratio of 3) (57), as low as 244 nM, could be obtained for thrombin, if the two probes were used to detect thrombin based on the specific fluorescent recognition of different TBA quadruplexes.

Both ThT and NMM become fluorescent in the presence of most G4, and the recent report by Mergny's group presented that the ThT fluorescence enhancement could be used as a diagnostic tool to screen the G4-forming sequences, but TBA was the only exception of the known G4-forming oligonucleotides (33). In our study, we demonstrated that ThT was capable of recognizing TBA with great fluorescence enhancement only in the conformation of parallel quadruplex, which was an effective complement to the investigation of using ThT as a high-throughput assay to predict G4 formation. It is noteworthy that, though the chair-like intramolecular anti-parallel quadruplex was well studied for TBA in the presence of thrombin or cations, such as K^+^ or Sr^2+^, there was hardly any report on the parallel quadruplex of TBA. Here, the results of ICD spectra and FRET experiments indicated that ThT was capable of inducing TBA to fold into a parallel quadruplex in the absence of cations, which was probably similar to the conformation of mutated TBA reported by the teams of Modesto Orozco and Ramon Eritja, an intermolecular parallel quadruplex composed of two strands (43). In this case, ThT could bound to the parallel TBA quadruplex mainly with the intercalation mode, which was beneficial to immobilizing ThT at a right angle and preserve the excited state, resulting in a great enhancement in ThT fluorescence intensity (34–36). However, when the parallel TBA quadruplex was transformed into anti-parallel conformation by K^+^ or thrombin, both ICD spectra and molecular docking simulation experiments showed that ThT bound to the anti-parallel quadruplex only in the groove region, as the two quartets of the anti-parallel TBA quadruplex did not offer an optimal site for ThT sequestration, there was almost no promotion in ThT emission intensity.

On the other hand, NMM exhibited highly selective fluorescent recognition of the anti-parallel TBA quadruplex with the end-stacking mode, through binding to the region of two T loops with strong π∼π interactions as well as the bottom quartet of G4 with intermolecular hydrogen bonds. However, for ThT-induced parallel TBA quadruplex, the two T loops were separated from each other, and then made it hard for NMM to bind with it, therefore, leading to almost no enhancement in NMM fluorescence intensity.

**Scheme 2.**
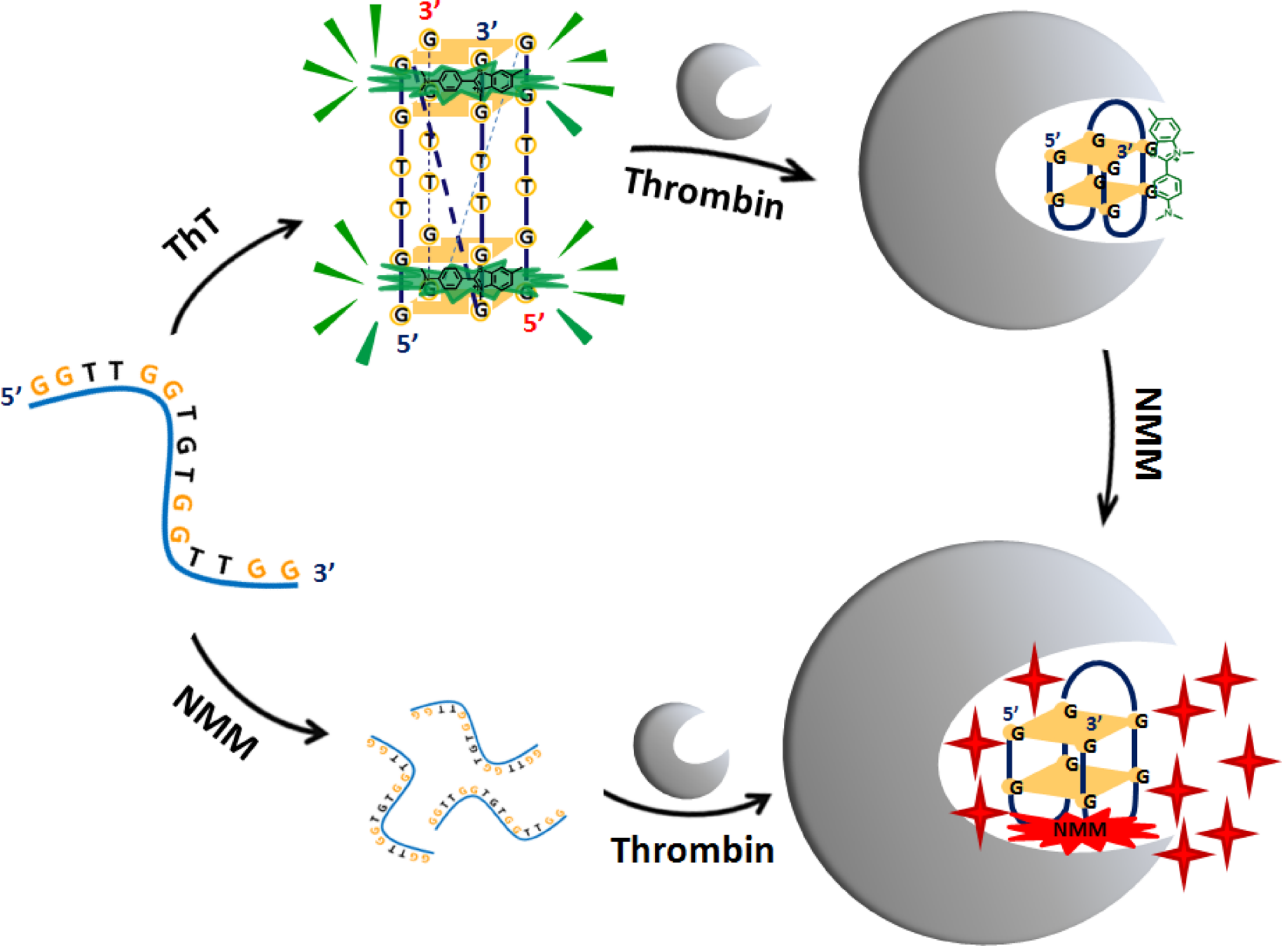
Schematic representation of selectively fluorescent recognition of the parallel and anti-parallel TBA G-quadruplexes by ThT and NMM, respectively.

ThT and NMM have been used as label-free turn-on fluorescent sensors for most G4-forming sequences, but for TBA, our study demonstrated that the emission enhancement of ThT or NMM was only obtained in the presence of responding parallel or anti-parallel TBA quadruplex, respectively. The highly selective recognition of TBA quadruplex with different topologies by the two probes was presented in Scheme 2, it may be useful to investigate the interactions between conformation-specific G4 and the associated proteins, and could also be applied in label-free fluorescent sensing of other biomolecules.

## SUPPLEMENTARY DATA

Supplementary Data are available at NAR Online.

SUPPLEMENTARY DATA

## References

[1] Burge S., Parkinson G.N., Hazel P., Todd A.K., Neidle S. (2006). Quadruplex DNA: sequence, topology and structure. Nucleic Acids Res..

[2] Lipps H.J., Rhodes D. (2009). G-quadruplex structures: in vivo evidence and function. Trends Cell Biol..

[3] Maizels N. (2006). Dynamic roles for G4 DNA in the biology of eukaryotic cells. Nat. Struct. Mol. Biol..

[4] Patel D.J., Phan A.T., Kuryavyi V. (2007). Human telomere, oncogenic promoter and 5′-UTR G-quadruplexes: diverse higher order DNA and RNA targets for cancer therapeutics. Nucleic Acids Res..

[5] Parkinson G.N., Lee M.P.H., Neidle S. (2002). Crystal structure of parallel quadruplexes from human telomeric DNA. Nature.

[6] Ying L., Green J.J., Li H., Klenerman D., Balasubramanian S. (2003). Studies on the structure and dynamics of the human telomeric G quadruplex by single-molecule fluorescence resonance energy transfer. Proc. Natl. Acad. Sci. U.S.A..

[7] Rachwal P.A., Brown T., Fox K.R. (2007). Effect of G-tract length on the topology and stability of intramolecular DNA quadruplexes. Biochemistry.

[8] Bugaut A., Balasubramanian S. (2008). A sequence-independent study of the influence of short loop lengths on the stability and topology of intramolecular DNA G-quadruplexes. Biochemistry.

[9] Kerwin S.M. (2000). G-quadruplex DNA as a target for drug design. Curr. Pharm. Des..

[10] Hong Y., Hauβler M., Lam J.W., Li Z., Sin K.K., Dong Y., Tong H., Liu J., Qin A., Renneberg R., Tang B.Z. (2008). Label-free fluorescent probing of G-quadruplex formation and real-time monitoring of DNA folding by a quaternized tetraphenylethene salt with aggregation-induced emission characteristics. Chem. Eur. J..

[11] Miyoshi D., Nakao A., Sugimoto N. (2003). Structural transition from antiparallel to parallel G-quadruplex of d(G4T4G4) induced by Ca^2+^. Nucleic Acids Res..

[12] Miyoshi D., Karimata H., Wang Z.M., Koumoto K., Sugimoto N. (2007). Artificial G-wire switch with 2,2′-bipyridine units responsive to divalent metal ions. J. Am. Chem. Soc..

[13] Sen D., Gilbert W. (1988). Formation of parallel four-stranded complexes by guanine-rich motifs in DNA and its implications for meiosis. Nature.

[14] Schaffitzel C., Berger I., Postberg J., Hanes J., Lipps H.J., Pluckthun A. (2001). In vitro generated antibodies specific for telomeric guanine-quadruplex DNA react with Stylonychia lemnae macronuclei. Proc. Natl. Acad. Sci. U.S.A..

[15] Siddiqui-Jain A., Grand C.L., Bearss D.J., Hurley L.H. (2002). Direct evidence for a G-quadruplex in a promoter region and its targeting with a small molecule to repress c-MYC transcription. Proc. Natl. Acad. Sci. U.S.A..

[16] Paeschke K., Simonsson T., Postberg J., Rhodes D., Lipps H.J. (2005). Telomere end-binding proteins control the formation of G-quadruplex DNA structures in vivo. Nat. Struct. Mol. Biol..

[17] Hershman S.G., Chen Q., Lee J.Y., Kozak M.L., Yue P., Wang L.S., Johnson F.B. (2008). Genomic distribution and functional analyses of potential G-quadruplex-forming sequences in Saccharomyces cerevisiae. Nucleic Acids Res..

[18] Fernando H., Sewitz S., Darot J., Tavare S., Huppert J.L., Balasubramanian S. (2009). Genome-wide analysis of a G-quadruplex-specific single-chain antibody that regulates gene expression. Nucleic Acids Res..

[19] Cahoon L.A., Seifert H.S. (2009). An alternative DNA structure is necessary for pilin antigenic variation in Neisseria gonorrhoeae. Science.

[20] Smith J.S., Chen Q., Yatsunyk L.A., Nicoludis J.M., Garcia M.S., Kranaster R., Balasubramanian S., Monchaud D., Teulade-Fichou M.P., Abramowitz L. (2011). Rudimentary G-quadruplex-based telomere capping in Saccharomyces cerevisiae. Nat. Struct. Mol. Biol..

[21] Eddy J., Vallur A.C., Varma S., Liu H., Reinhold W.C., Pommier Y., Maizels N. (2011). G4 motifs correlate with promoter-proximal transcriptional pausing in human genes. Nucleic Acids Res..

[22] Chen C.Y., Wang Q., Liu J.Q., Hao Y.H., Tan Z. (2011). Contribution of telomere G-quadruplex stabilization to the inhibition of telomerase-mediated telomere extension by chemical ligands. J. Am. Chem. Soc..

[23] Rodriguez R., Miller K.M., Forment J.V., Bradshaw C.R., Nikan M., Britton S., Oelschlaegel T., Xhemalce B., Balasubramanian S., Jackson S.P. (2012). Small-molecule–induced DNA damage identifies alternative DNA structures in human genes. Nat. Chem. Biol..

[24] Zheng K.W., Xiao S., Liu J.Q., Zhang J.Y., Hao Y.H., Tan Z. (2013). Co-transcriptional formation of DNA:RNA hybrid G-quadruplex and potential function as constitutional cis element for transcription control. Nucleic Acids Res..

[25] Zhang D., Deng M., Xu L., Zhou Y., Yuwen J., Zhou X. (2009). The sensitive and selective optical detection of mercury(II) ions by using a phosphorothioate DNAzyme strategy. Chem. Eur. J..

[26] Dash J., Shirude P.S., Hsu S.-T.D., Balasubramanian S. (2008). Diarylethynyl amides that recognize the parallel conformation of genomic promoter DNA G-quadruplexes. J. Am. Chem. Soc..

[27] Hu D., Pu F., Huang Z., Ren J., Qu X. (2010). A quadruplex-based, label-free, and real-time fluorescence assay for RNase H activity and inhibition. Chem. Eur. J..

[28] Yan S., Huang R., Zhou Y., Zhang M., Deng M., Wang X., Weng X., Zhou X. (2011). Aptamer-based turn-on fluorescent four-branched quaternary ammonium pyrazine probe for selective thrombin detection. Chem. Commun..

[29] Tian T., Xiao H., Zhang Z., Long Y., Peng S., Wang S., Zhou X., Liu S., Zhou X. (2013). Sensitive and convenient detection of microRNAs based on cascade amplification by catalytic DNAzymes. Chem. Eur. J..

[30] Mohanty J., Barooah N., Dhamodharan V., Harikrishna S., Pradeepkumar P.I., Bhasikuttan A.C. (2013). Thioflavin T as an efficient inducer and selective fluorescent sensor for the human telomeric G-quadruplex DNA. J. Am. Chem. Soc..

[31] Tong L.L., Li L., Chen Z., Wang Q., Tang B. (2013). Stable label-free fluorescent sensing of biothiols based on ThT direct inducing conformation-specific G-quadruplex. Biosens. Bioelectron..

[32] Gabelica V., Maeda R., Fujimoto T., Yaku H., Murashima T., Sugimoto N., Miyoshi D. (2013). Multiple and cooperative binding of fluorescence light-up probe thioflavin T with human telomere DNA G-quadruplex. Biochemistry.

[33] Renaud de la Faverie A., Guedin A., Bedrat A., Yatsunyk L.A., Mergny J.L. (2014). Thioflavin T as a fluorescence light-up probe for G4 formation. Nucleic Acids Res..

[34] Vassar P.S., Culling C.F. (1959). Fluorescent stains, with special reference to amyloid and connective tissues. Arch Pathol..

[35] Wolfe L.S., Calabrese M.F., Nath A., Blaho D.V., Miranker A.D., Xiong Y. (2010). Protein-induced photophysical changes to the amyloid indicator dye thioflavin T. Proc. Natl. Acad. Sci. U.S.A..

[36] Biancalana M., Koide S. (2010). Molecular mechanism of Thioflavin-T binding to amyloid fibrils. Biochimica et biophysica acta.

[37] Fenton J.W. (1981). Thrombin specificity. Ann. N.Y. Acad. Sci..

[38] Shuman M.A. (1986). Thrombin-cellular interactions. Ann. N.Y. Acad. Sci..

[39] Russo Krauss I., Merlino A., Giancola C., Randazzo A., Mazzarella L., Sica F. (2011). Thrombin-aptamer recognition: a revealed ambiguity. Nucleic Acids Res..

[40] Marson G., Palumbo M., Sissi C. (2012). Folding versus charge: understanding selective target recognition by the thrombin aptamers. Curr. Pharm. Des..

[41] De Rache A., Kejnovska I., Vorlickova M., Buess-Herman C. (2012). Elongated thrombin binding aptamer: a G-quadruplex cation-sensitive conformational switch. Chem. Eur. J..

[42] Kankia B.I., Marky L.A. (2001). Folding of the thrombin aptamer into a G-quadruplex with Sr^2+^: stability, heat, and hydration. J. Am. Chem. Soc..

[43] Avino A., Portella G., Ferreira R., Gargallo R., Mazzini S., Gabelica V., Orozco M., Eritja R. (2014). Specific loop modifications of the thrombin-binding aptamer trigger the formation of parallel structures. FEBS.

[44] Arthanari H., Basu S., Kawano T.L., Bolton P.H. (1998). Fluorescent dyes specific for quadruplex DNA. Nucleic Acids Res..

[45] Ren J., Chaires J.B. (1999). Sequence and structural selectivity of nucleic acid binding ligands. Biochemistry.

[46] Hu D., Huang Z., Pu F., Ren J., Qu X. (2011). A label-free, quadruplex-based functional molecular beacon (LFG4-MB) for fluorescence turn-on detection of DNA and nuclease. Chem. Eur. J..

[47] Haider S., Neidle S. (2010). Molecular modeling and simulation of G-quadruplexes and quadruplex-ligand complexes. Methods Mol. Biol..

[48] Morris G.M., Goodsell D.S., Halliday R.S., Huey R., Hart W.E., Belew R.K., Olson A.J. (1998). Automated docking using a Lamarckian genetic algorithm and an empirical binding free energy function. J. Comput. Chem..

[49] Morris G.M., Goodsell D.S., Huey R., Olson A.J. (1996). Distributed automated docking of flexible ligands to proteins: Parallel applications of AutoDock 2.4. J. Comput. Aided Mol. Des..

[50] Dash J., Shirude P.S., Hsu S.-T.D., Balasubramanian S. (2008). Diarylethynyl amides that recognize the parallel conformation of genomic promoter DNA G-quadruplexes. J. Am. Chem. Soc..

[51] Yamashita T., Uno T., Ishikawa Y. (2005). Stabilization of guanine quadruplex DNA by the binding of porphyrins with cationic side arms. Bioorg. Med. Chem..

[52] Huey R., Morris G.M., Olson A.J., Goodsell D.S. (2007). A semiempirical free energy force field with charge-based desolvation. J. Comput. Chem..

[53] Estiu G., Suarez D., Merz K.M. (2006). Quantum mechanical and molecular dynamics simulations of ureases and Zn beta-lactamases. J. Comput. Chem..

[54] Takishima K., Suga T., Mmiya G. (1988). The structure of jack bean urease the complete amino acid sequence, limited proteolysis and reactive cysteine residues. Eur. J. Biochem..

[55] Glbwka M.L., Martynowski D., Kozfowska K. (1999). Stacking of six-membered aromatic rings in crystals. J. Mol. Struct..

[56] Burley S.K., Petsko G.A. (1985). Aromatic-aromatic interaction: a mechanism of protein structure stabilization. Science.

[57] Lin Y.W., Liu C.W., Chang H.T. (2011). Fluorescence detection of mercury (II) and lead (II) ions using aptamer/reporter conjugates. Talanta.

