# Supplementary material for: Selective recognition of parallel and anti-parallel thrombin-binding aptamer G-quadruplexes by different fluorescent dyes
Source: Nucleic Acids Res. 2014 Sep 22;42(18):11612–21. doi: 10.1093/nar/gku833 (PMC4191408; doi:10.1093/nar/gku833)
Supplement: SUPPLEMENTARY DATA [file supp_42_18_11612__index.html]

Selective recognition of parallel and anti-parallel thrombin-binding aptamer G-quadruplexes by different fluorescent dyes — Selective recognition of parallel and anti-parallel thrombin-binding aptamer G-quadruplexes by different fluorescent dyes — SUPPLEMENTARY DATA 

# Selective recognition of parallel and anti-parallel thrombin-binding aptamer G-quadruplexes by different fluorescent dyes

## SUPPLEMENTARY DATA

**Files in this Data Supplement:**

- SUPPLEMENTARY DATA
