# Supplementary material for: Selective recognition of parallel and anti-parallel thrombin-binding aptamer G-quadruplexes by different fluorescent dyes
Source: Nucleic Acids Res. 2014 Sep 22;42(18):11612–21. doi: 10.1093/nar/gku833 (PMC4191408; doi:10.1093/nar/gku833)

Supplementary Data

Selective Recognition of Parallel and Anti-parallel Thrombin-Binding Aptamer G-quadruplexes by Different Fluorescent Dyes

Dan Zhao[1], Xiongwei Dong[1], Nan Jiang[1], Dan Zhang[1,*] and Changlin Liu[1,*]

[1] Key Laboratory of Pesticide and Chemical Biology, Ministry of Education, and School of Chemistry, Central China Normal University, Wuhan 430079, China.

Tel: +86 27 6786 7273; Fax: +86 27 6786 7273; Email: danzhang@mail.ccnu.edu.cn; correspondence may also be addressed to liuchl@mail.ccnu.edu.cn

Figure S1. UV-vis titration of ThT (10 μM) with TBA in 10 mM Tris-HCl buffer, pH 7.2. The arrows indicate an increment in TBA concentration from 0 to 45 μM.

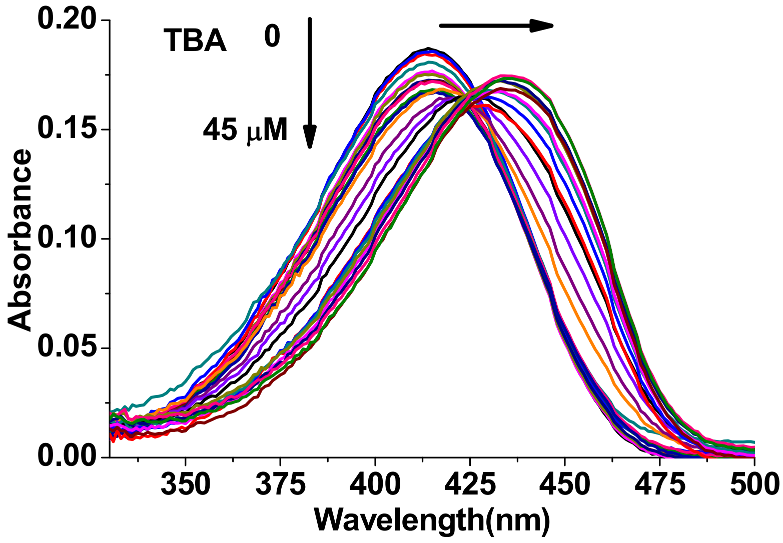

Scheme S1. Schematic representation of the folding of parallel and anti-parallel TBA quadruplexes induced by K^+ and ThT, respectively.

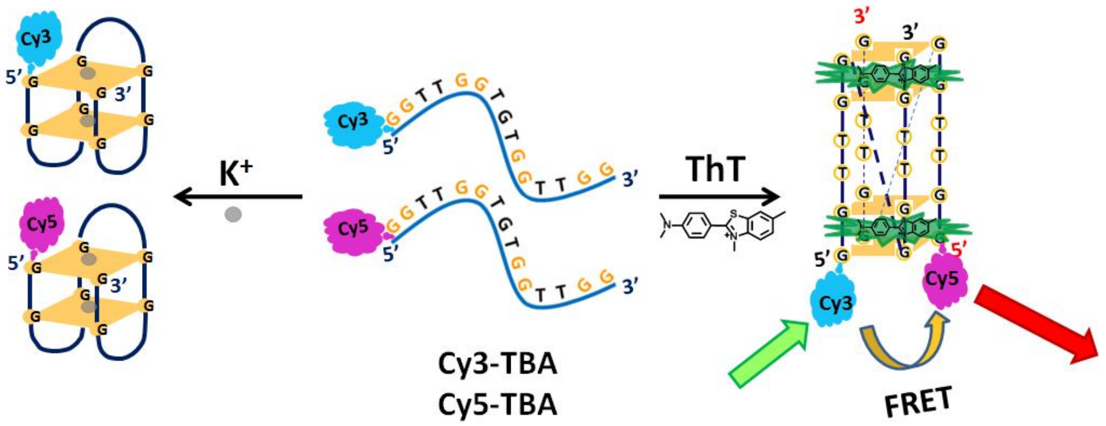

Figure S2. a) Fluorescence titration of labelled TBA with various concentrations of ThT. The arrow from bottom to top represents increments of ThT from 0 to 400 μM. Insert: Plot the emission intensity at 667 nm ($I_{667\ nm}$) against the concentration of ThT. b) Fluorescence titration of labelled TBA with various concentrations of K^+ (0 ~ 500 mM). c) Fluorescence titration of Cy3-TBA (1 μM) with ThT. The arrow from top to bottom represents increments of ThT from 0 to 400 μM. d) Fluorescence titration of labelled TBA2G with various concentrations of K^+. The arrow from bottom to top represents increments of ThT from 0 to 0.8 M. Insert: Plot the emission intensity at 667 nm against the concentration of K^+. The labelled TBA/TBA2G used here included 1μM Cy3-TBA/TBA2G and 1μM Cy5-TBA/TBA2G. λ_{ex} = 544 nm.

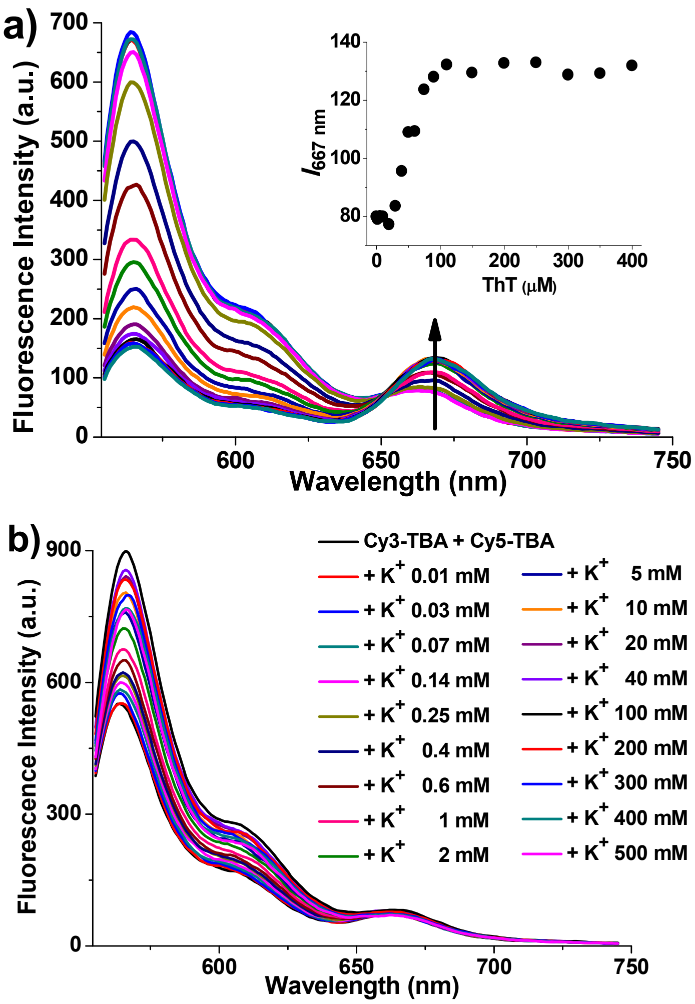

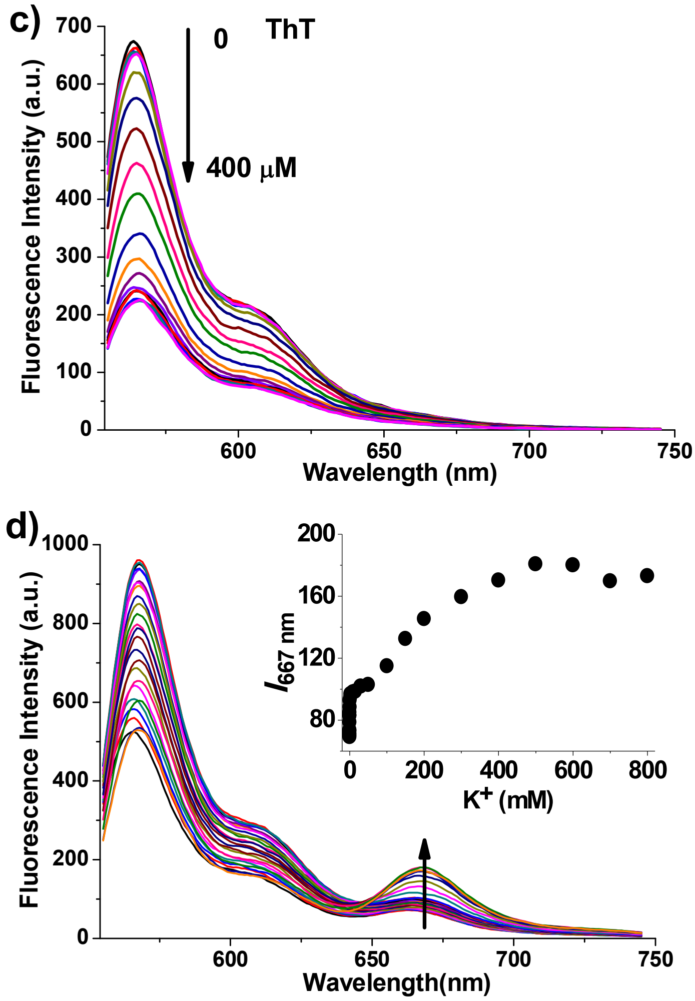

c)
0
ThT
400 μM
Fluorescence Intensity (a.u.)
Wavelength (nm)
d)
Fluorescence Intensity (a.u.)
Wavelength(nm)
I667 nm
K+ (mM)

Figure S3. Normalized melting curves for TBA 15 μM (a), 30 μM (b) and 45 μM (c) in the presence of 10, 15, 20, 30 or 40 equiv of ThT, respectively. d) Linear response of Tm vs. “r” was recorded. r=[ThT]/[TBA]

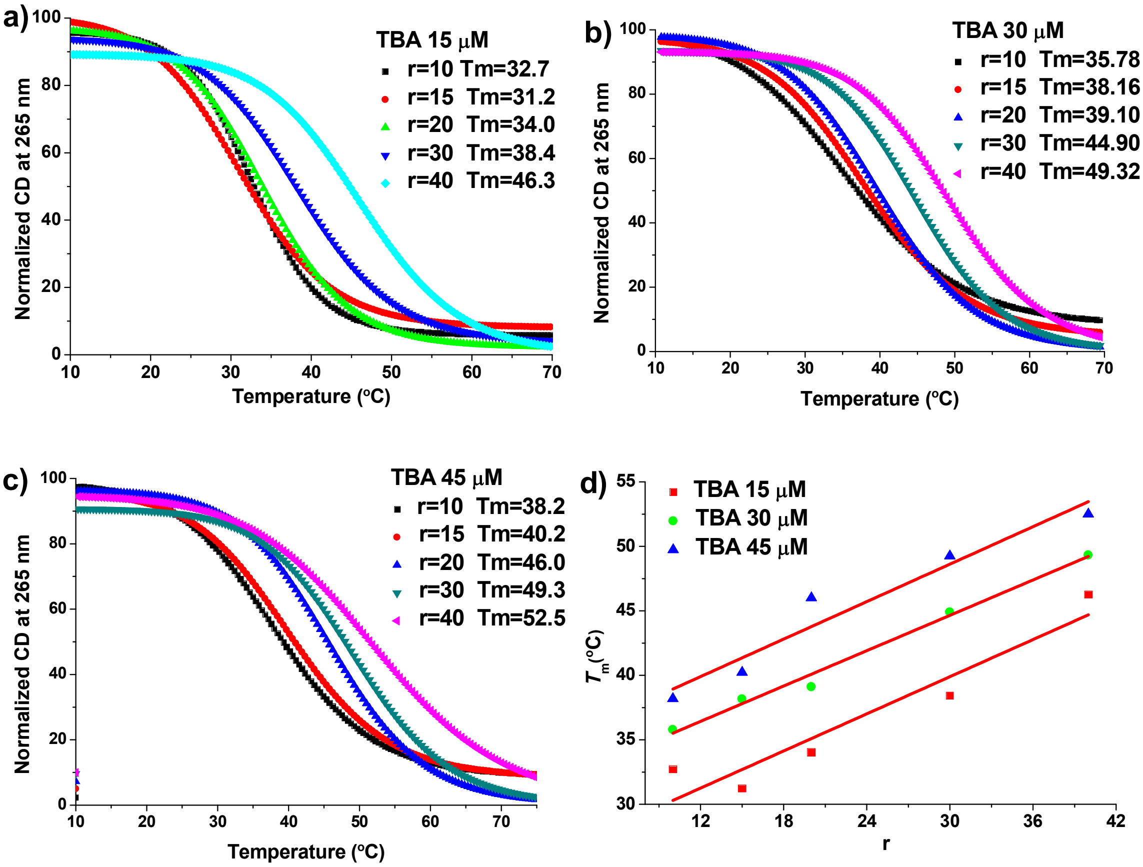

Figure S4. a) CD spectra recorded for TBA (3 μM) in water with ThT in the absence of metal ions, r = [ThT]/[TBA]. The arrow indicates increments in r values from 0 to 80. b) Fluorescence titration of ThT (5 μM in water) with various concentrations of TBA, λ_{ex} = 420 nm. The arrow indicates an increment in TBA concentration from 0 to 450 nM.

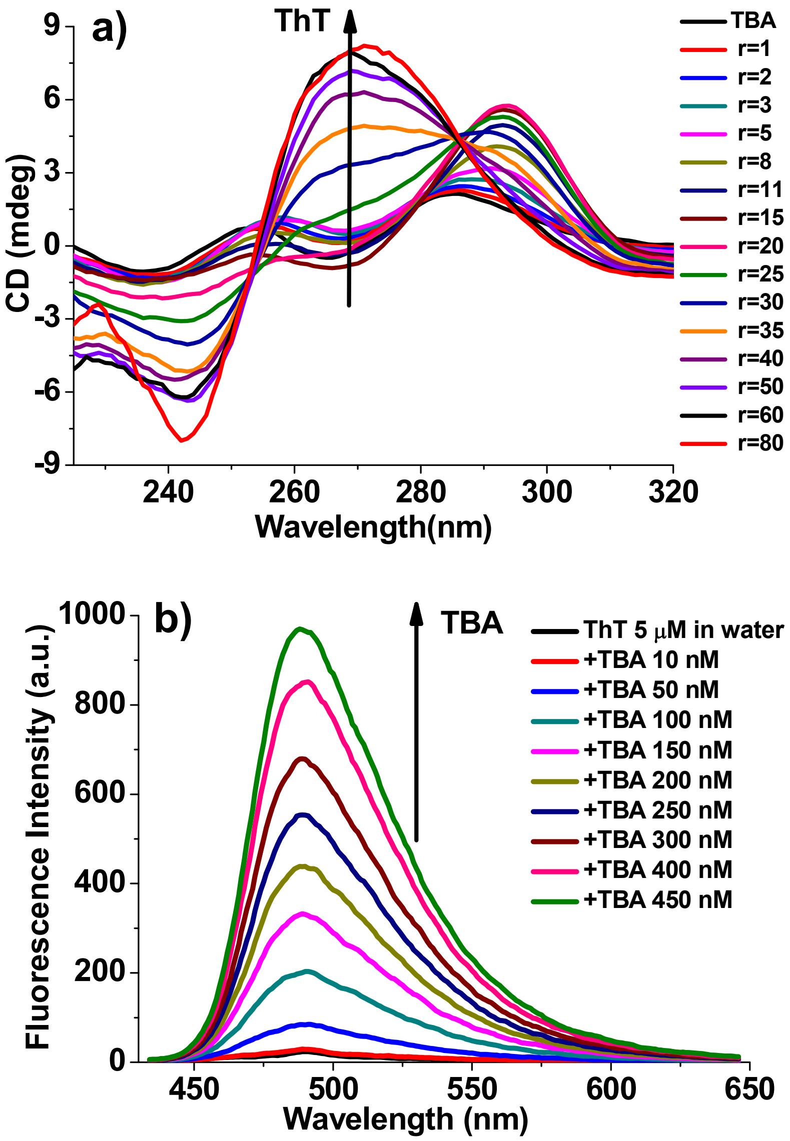

Figure S5. CD and fluorescence titration of TBA or the mixture of TBA and ThT with K^+ in 10 mM Tris-buffer, pH 7.2. a) CD spectra recorded for TBA (3 μM) with addition of K^+. The arrow from bottom to top indicates increments in K^+ concentrations from 0 to 300 μM. b) The parallel G-quadruplex formed with TBA (3 μM) in the presence of 50 equiv of ThT was translated into the anti-parallel G-quadruplex upon the addition of K^+ from 0 to 9 mM. c) The emission intensity of ThT (5 μM) in the presence of 1 μM TBA decreased with the addition of K^+ from 0 to 300 mM.

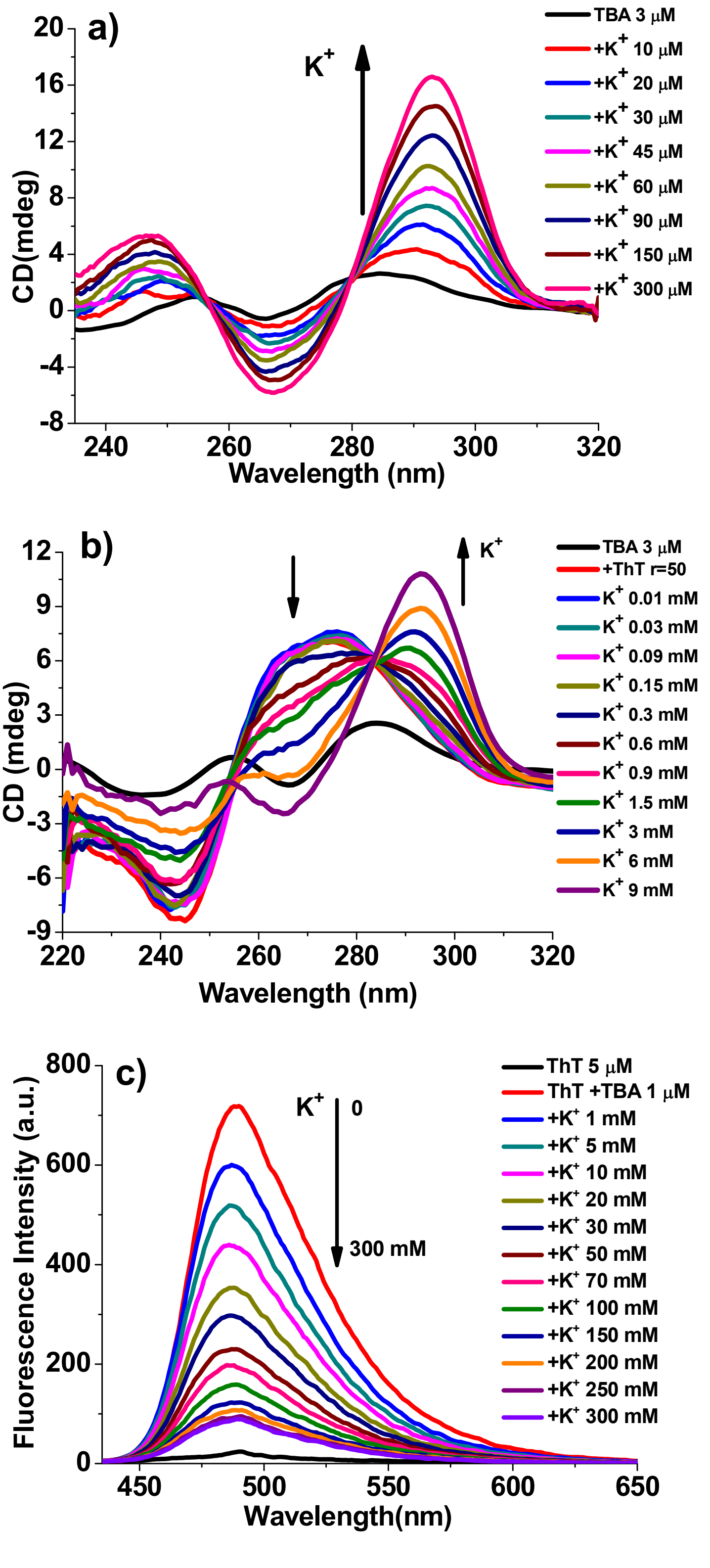

Figure S6. CD spectra recorded for TBA (3 µM) in 10 mM Tris-buffer, pH 7.2, with Sr^{2+} ions (from 0 to 90 µM) and EDTA (900 µM). The arrow from bottom to top indicates increments in Sr^{2+} concentrations from 0 to 90 µM

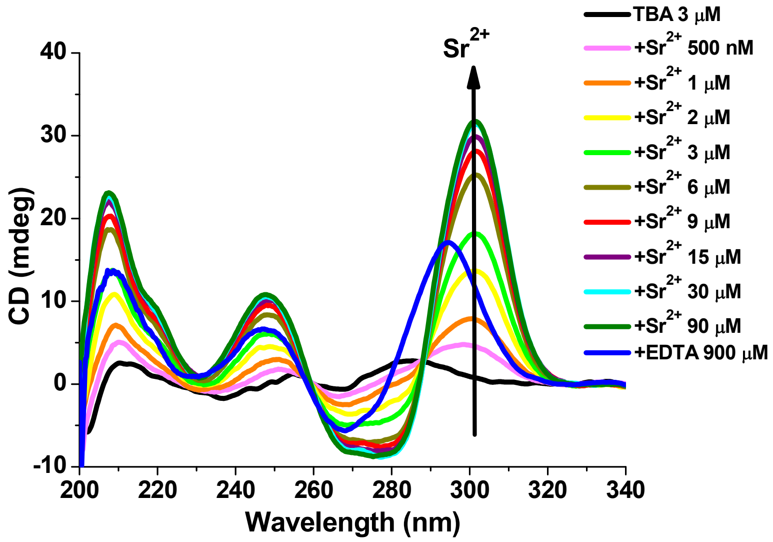

Figure S7. a) CD spectra recorded for TBA (3 μM) upon titration with 1 ~ 20 equiv of NMM in the absence of cationic ions. b) Fluorescence spectra of NMM (10 μM) on titration with TBA (0 ~ 4 μM). c, d) Fluorescence spectra of NMM (10 μM) on titration with K^+ (0 ~ 300 mM, c) or Sr^{2+} (0 ~ 100 μM, d) in the presence of TBA (2 μM in c and 4 μM in d). All experiments were carried out in 10 mM Tris-buffer, pH 7.2, and the excitation wavelength was set at 400 nm.

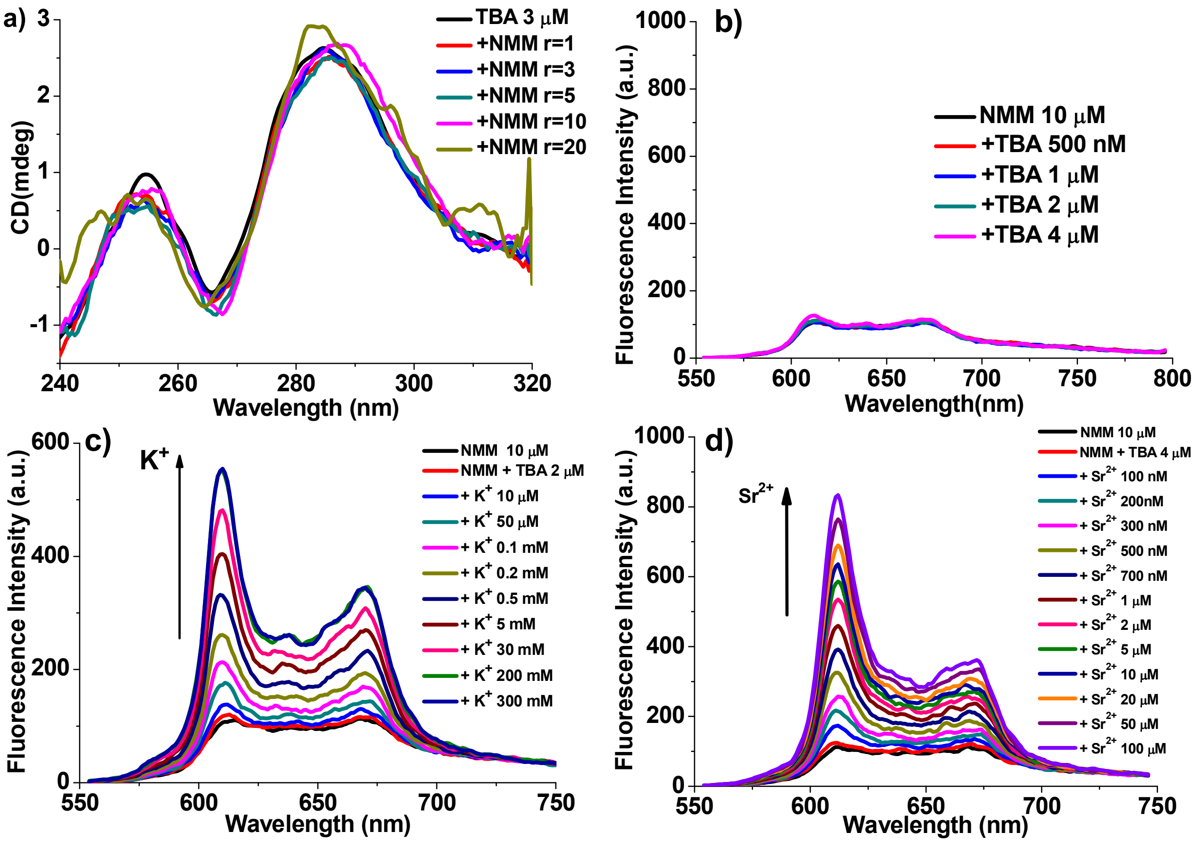

Figure S8. Fluorescence spectra of the mixed solution, 5 μM ThT and 10 μM NMM, in the presence of TBA (4 μM) upon the addition of K^{+} (a) or Sr^{2+} (b). The arrows indicate increments in K^{+} concentrations from 0 to 400 mM (a), and in Sr^{2+} from 0 to 100 μM (b). All titration were carried out in 10 mM Tris-buffer, pH 7.2. The excitation wavelength of the samples were set at 410 nm.

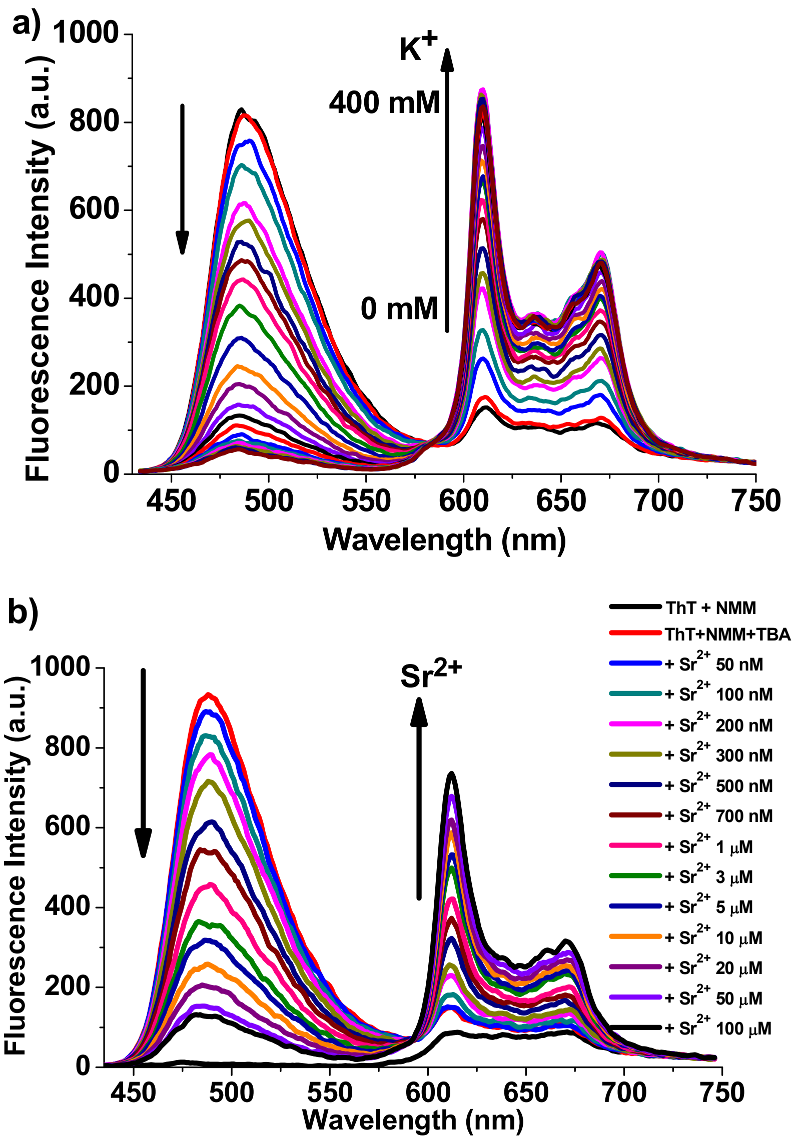

Figure S9. a) CD spectra titration of TBA (3 μM) with various concentrations of Thrombin (Tmb) in the presence of ThT (120 μM). The arrows indicate increments in r'' values from 0 to 20. (r'' = [Tmb]/[TBA]). b, c) Fluorescence spectra of ThT (b) or NMM (c) on titration with different concentrations of thrombin in the presence of TBA.

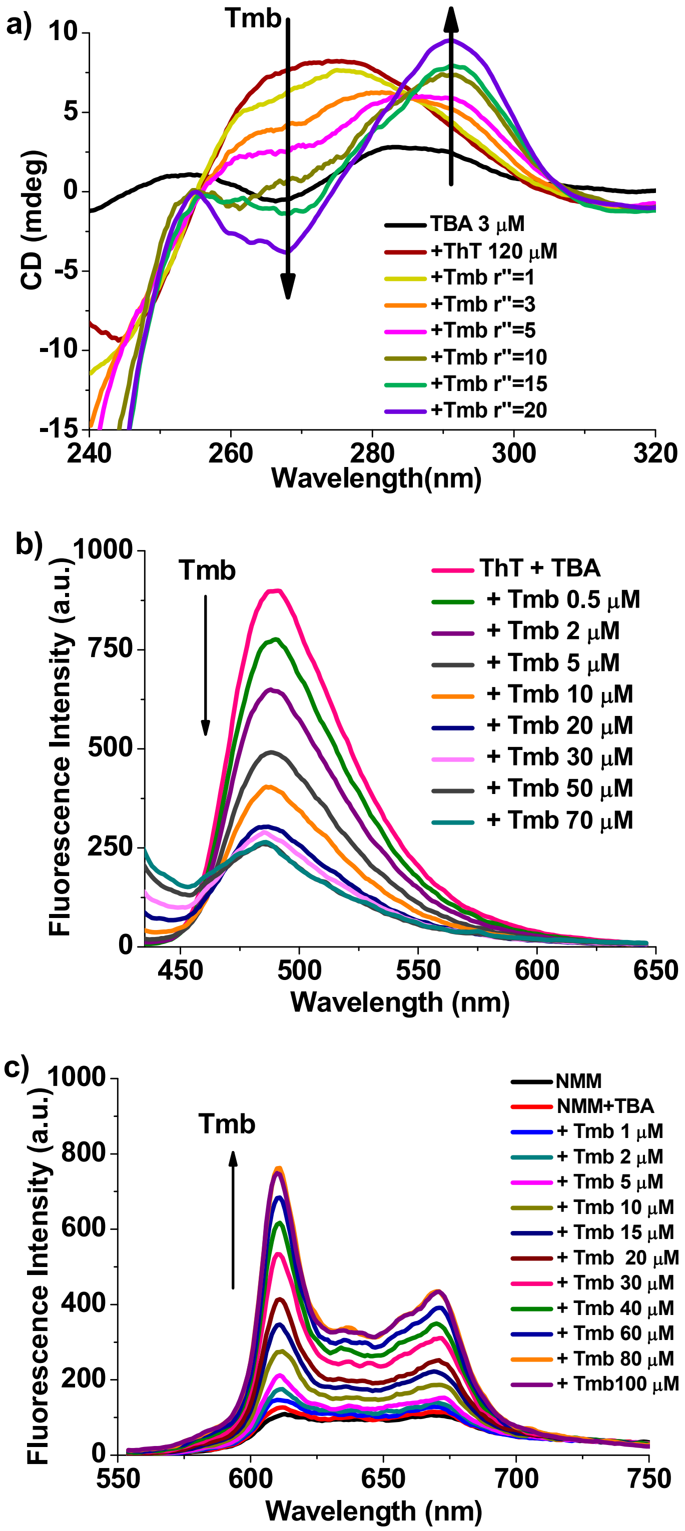

Supplement: SUPPLEMENTARY DATA [file supp_gku833_nar-01508-f-2014-File002.pdf]
